# Pattern of inbreeding depression, condition dependence, and additive genetic variance in Trinidadian guppy ejaculate traits

**DOI:** 10.1002/ece3.870

**Published:** 2013-11-08

**Authors:** Clelia Gasparini, Alessandro Devigili, Ryan Dosselli, Andrea Pilastro

**Affiliations:** Department of Biology, University of PadovaPadova, Italy

**Keywords:** Genetic benefits, good sperm models, lek paradox, polyandry, sperm competition

## Abstract

In polyandrous species, a male's reproductive success depends on his fertilization capability and traits enhancing competitive fertilization success will be under strong, directional selection. This leads to the prediction that these traits should show stronger condition dependence and larger genetic variance than other traits subject to weaker or stabilizing selection. While empirical evidence of condition dependence in postcopulatory traits is increasing, the comparison between sexually selected and ‘control’ traits is often based on untested assumption concerning the different strength of selection acting on these traits. Furthermore, information on selection in the past is essential, as both condition dependence and genetic variance of a trait are likely to be influenced by the pattern of selection acting historically on it. Using the guppy (*Poecilia reticulata*), a livebearing fish with high levels of multiple paternity, we performed three independent experiments on three ejaculate quality traits, sperm number, velocity, and size, which have been previously shown to be subject to strong, intermediate, and weak directional postcopulatory selection, respectively. First, we conducted an inbreeding experiment to determine the pattern of selection in the past. Second, we used a diet restriction experiment to estimate their level of condition dependence. Third, we used a half-sib/full-sib mating design to estimate the coefficients of additive genetic variance (CV_A_) underlying these traits. Additionally, using a simulated predator evasion test, we showed that both inbreeding and diet restriction significantly reduced condition. According to predictions, sperm number showed higher inbreeding depression, stronger condition dependence, and larger CV_A_ than sperm velocity and sperm size. The lack of significant genetic correlation between sperm number and velocity suggests that the former may respond to selection independently one from other ejaculate quality traits. Finally, the association between sperm number and condition suggests that this trait may mediate the genetic benefits of polyandry which have been shown in this species.

## Introduction

Sexual selection is a pervasive evolutionary force and has been shown to be, on average, stronger (Kingsolver et al. 2001) and more constant (Hoekstra et al. 2001) than natural selection. This is because a male can improve his reproductive success only at the expenses of other males, hence increasing the variance in fitness and hence the strength of selection (Shuster and Wade 2003). Directional sexual selection, as initially proposed by Darwin (1859), is expected to drive trait exaggeration up to the point at which its contribution to reproductive fitness is counterbalanced by its survival cost, or, in other words, at which sexual selection is balanced by natural selection (Andersson 1994). This implies that the trait becomes increasingly costly and if its cost is lower for high-quality males (Zahavi 1975; Andersson 1986; Grafen 1990), condition dependence is expected to evolve (Iwasa et al. 1991; Rowe and Houle 1996). Condition has been defined as the heritable component of the resources available to an individual for allocation to the production and maintenance of fitness-related traits (Rowe and Houle 1996; Tomkins et al. 2004). This has lead to the general prediction that traits that are under strong and persistent directional selection (such as sexually selected traits) should show stronger condition dependence than traits that are exposed to weaker or to stabilizing selection (Rowe and Houle 1996), because allocating more resources to such traits does not increase fitness (Delcourt and Rundle 2011). Condition dependence is central for the so-called good genes models of sexual selection (Grafen 1990; Iwasa et al. 1991; Rowe and Houle 1996) and has also important implications for the maintenance of genetic variation in fitness-related traits (Houle 1998). What maintains the genetic variation in natural populations in the presence of strong selection is a central question in evolutionary biology (Walsh and Blows 2009). Indeed, genetic variation in individual traits is ubiquitous (Lynch and Walsh 1998), and most paradoxically, this variation appears to be larger the closer a trait is to fitness, that is, subject to stronger selection (Houle 1992; Pomiankowski and Møller 1995). Condition dependence offers an answer to this paradox, as the resources an individual can allocate to the trait are likely to be influenced by large part of the genome. If a trait, because of its association with condition, is affected by most of the genome, it is more likely to be targeted by deleterious mutations (Rowe and Houle 1996) and to be affected by multivariate genetic constraints (Walsh and Blows 2009), two processes that will both contribute to the maintenance of genetic variation.

Despite being apparently straightforward, testing experimentally the condition dependence of sexually selected traits can be complicated because the effect of male condition on sexually selected traits requires to be compared with other traits for which the strength (and the shape) of selection is known (Cotton et al. 2004). If it can be assumed that in general, naturally selected traits are subject to weaker or otherwise stabilizing selection, many nonsexually selected traits are also close to fitness and show condition dependence, like, for example, most life-history traits (Rowe and Houle 1996; Houle 1998). Although most recent studies include nonsexual traits as a control, the strength of selection on these traits has been rarely estimated directly, and it is often only assumed (see Delcourt and Rundle 2011 for a review). If strength of selection is assumed, it cannot be used to estimate the relationship between the strength of condition dependence and selection. Although in principle sexual selection is expected to be strong and directional, this may not always be the case. For example, males often exhibit multiple sexual traits (Candolin 2003), and the expression of a particular combination of these traits, rather than the overall (or individual) expression of individual traits may be important (Kunzler and Bakker 2001; Hine et al. 2002). Furthermore, the strength and direction of the selection acting on a trait can vary according to female preference (Brooks and Endler 2001b). Finally, sexual selection may have nondirectional components that need to be estimated (Zajitschek and Brooks 2008; Gasparini and Pilastro 2011). A final complication is that the strength and the shape of selection presently acting on a population may differ from that acting in the past, possibly obscuring the relationship between current selection on a trait and its level of condition dependence and genetic variance (Delcourt and Rundle 2011).

Sexual selection can continue after mating in those cases in which females mate with several males during a single reproductive episode, a taxonomically widespread reproductive strategy (Birkhead and Møller 1998; Simmons 2001) with far-reaching evolutionary consequences (Birkhead and Pizzari 2002; Simmons 2005; Kvarnemo and Simmons 2013). One of the most prominent implications of polyandry is that it promotes postcopulatory sexual selection in the form of sperm competition, where the ejaculates of two or more males compete to fertilize the same set of ova (Birkhead and Møller 1998). Males producing ejaculates with greater fertilization success are favored under these conditions (Birkhead et al. 2009). As with other sexually selected traits, directional selection exerted by polyandry on male postcopulatory traits is expected to increase the investment in traits associated with fertilization success and condition dependence is expected to evolve as the cost of these traits increases (Knell and Simmons 2010). While empirical tests of condition dependence of sexual traits have been mainly performed in a precopulatory context (reviewed in Cotton et al. 2004; Tomkins et al. 2004; Kotiaho et al. 2008; Radwan 2008), studies on postcopulatory sexual traits are far less numerous. In *Onthophagus* beetles, for example, condition shows a positive genetic correlation with sperm size, the primary predictor of postcopulatory success in these species (Simmons and Kotiaho 2002, 2007). In contrast, diet restriction has little effect of condition on sperm production in the butterfly *Bicyclus anynana* (Lewis and Wedell 2007), whereas it affects sperm number but not sperm size in the moth *Plodia interpunctella* (Gage and Cook 1994). In the first species, sperm competition level is low (Brakefield et al. 2001), whereas in the second, the transfer of large numbers of fertile sperm is associated with higher fertilization success under sperm competition (Lewis et al. 2011). These results may therefore be in agreement with the theoretical predictions that condition dependence in postcopulatory sexual traits reflects the strength of selection acting on those traits. However, strength of selection on sexual and nonsexual traits has been rarely measured directly (Delcourt and Rundle 2011) and, as far as we are aware, never in a postcopulatory context.

We compared the levels of additive genetic variance and condition dependence in three ejaculate traits using the guppy (*Poecilia reticulata*), a species whose postcopulatory sexual selection mechanisms have been studied in detail (see Evans and Pilastro 2011 for a review). This species is characterized by a resource-free mating system in which males provide no direct benefits to females (Pilastro et al. 2008), yet females mate with several males at each breeding cycle (Houde 1997), resulting in one of the highest levels of multiple paternity reported for any vertebrate species (Neff et al. 2008). Multiply mated females produce higher quality offspring (Evans and Magurran 2000; Ojanguren et al. 2005; Barbosa et al. 2012), suggesting that sperm competition may mediate genetic benefits to the female. The experimental manipulation of the number of competing sperm from two males, using artificial insemination revealed that sperm competition success is positively correlated with the number of sperm inseminated and, once sperm number is controlled for, with sperm velocity. Linear selection on sperm number is about as twice as stronger (standardized multiple linear coefficient, *β* = 0.67, 95% C.I. = 0.37–0.96) than that on sperm velocity (*β* = 0.32, 95% C.I. = 0.09–0.55). In contrast, the length of the sperm and of its components (sperm head, midpiece, and tail) is relatively unimportant in determining paternity success (Boschetto et al. 2011). If a trait's exaggeration and its condition dependence are proportional to the strength of directional selection acting on it (Houle 1998), we expect to unravel larger condition dependence and additive genetic variation in sperm number compared with sperm velocity and size. We tested these two predictions using three independent experiments.

The results by Boschetto et al. (2011) give an estimation of actual selection strength on sperm number, velocity, and size. However, condition dependence is the result of an evolutionary process determined by the selection pattern acting historically on the trait. In the first experiment, we therefore performed an inbreeding experiment to test whether sperm number has also been subjected in the past to stronger directional selection than sperm velocity and size. Inbreeding depression, or directional dominance, is the reduction in trait values in inbred individuals as compared with their outbred counterparts (Charlesworth and Charlesworth 1999). When a trait is subject to directional selection, mutations with a positive effect on trait expression should be fixed, whereas dominant mutations with deleterious effect on the trait should be removed by selection, and partially recessive deleterious alleles will be maintained at low frequency, which provides directionality (Lynch and Walsh 1998). Along with theoretical predictions, empirical data indicate that traits closely associated with fitness have significantly higher dominance components than traits more distantly related to fitness (Crnokrak and Roff 1995; Ala-Honkola et al. 2013). We therefore compared the mean phenotypic values of sperm number, velocity, and size in outbred male guppies with that of males after two generations of full-sib matings (inbreeding coefficient, *f* = 0.375).

In the second experiment, we tested whether sperm number, velocity, and size show a different degree of condition dependence by manipulating the resources available to males in a diet restriction experiment. To this end, we randomly assigned two groups of adult males to either an ad libitum (i.e., nonrestricted) or a restricted food diet treatment. After 6 weeks of diet treatment, we estimated the effect of diet restriction on sperm number, velocity, and size. Previous diet restriction experiments conducted on another feral population provided contrasting results, ranging from largely no effect of diet on sperm number, velocity, and size (Devigili et al. 2013) to all these traits being affected by diet restriction (Rahman et al. 2013). In the first two experiments, we directly estimated male condition by measuring their ability to escape a simulated predator (Evans and Magurran 2000).

In the third experiment, we conducted a half-sibs/full-sibs quantitative genetic analyses (Falconer and Mackay 1996; Lynch and Walsh 1998) to directly estimate the among-males genetic variability in the three sperm traits. We used a standardized measure of additive genetic variance to compare the genetic variability in sperm number, velocity, and size (Houle 1992), to test the prediction that traits subject to stronger selection show larger genetic variance, as compared to traits subject to weaker (or stabilizing) selection. Altogether, these three experiments will allow to shed light on the evolution of ejaculate quality traits in a vertebrate species with high levels of sperm competition.

## Materials and Methods

### Study population and its maintenance

**Figure 1:**
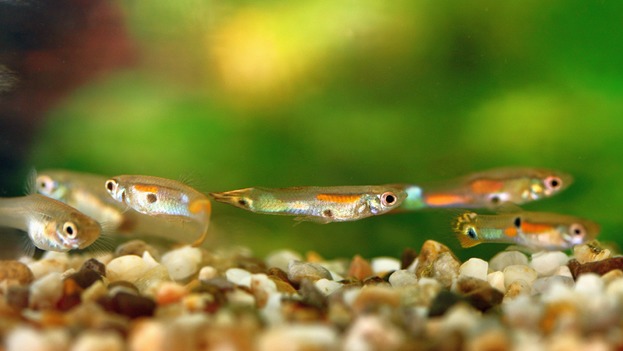
Male guppies (*Poecilia reticulata*) from the Lower Tacarigua (Trinidad) population used in this study.

The guppies used in this experiment were descendants of wild-caught fish collected in 2002 from the lower part of Tacarigua River in Trinidad (Trinidad national grid reference: PS 787,804; coordinates: N10°40.736′, W061°19.168, Fig. 1). Laboratory stock and all experimental fish were maintained under controlled temperature and lighting conditions (26 ± 1°C; 12: 12 h light/dark cycle) and fed twice daily on a mixed diet of brine shrimp nauplii (*Artemia salina*) and commercially prepared dry food (DuplaRin). Males and females used in the experiments derived from large stock tanks (150 L), each containing approx. 50 individuals of each sex that were allowed to breed freely. Effective population size was constantly kept above 500 individuals. Haphazardly, once or twice a year, 50–100 virgin females were mated singularly with an equal number of individual males and all the produced offspring was recruited in the stock population in order to preserve genetic variation. Indeed, the number of alleles and the heterozygosity at three neutral microsatellite loci used for paternity analysis did not show signs of decrease from 2002 to 2011 (Evans et al. 2003; Gasparini et al. 2010a; Boschetto et al. 2011; Gasparini and Pilastro 2011). Virgin females (4 months old) were reared in single-sex groups under the same temperature, light, and food regimes as the stock fish.

### General methods

#### Measurement of ejaculate traits and condition

Prior to ejaculate collection, males were anesthetized in a water bath containing tricaine mesylate (MS222) and photographed using a digital camera (Nikon Coolpix 4300, Nikon Corporation, Tokyo, Japan) with a millimeter ruler for calibration. Image analysis software (Image Tool: http://ddsdx.uthscsa.edu/dig/download.html) was used to estimate the standard length (distance from the snout to the end of the tail, SL). After the photograph, each anesthetized male was immediately placed on a glass slide under a stereomicroscope. The gonopodium was swung forward, and gentle pressure was applied to the side of the abdomen, close to the base of the gonopodium, to release sperm that are packaged in bundles.

Six sperm bundles were used for the sperm velocity analyses (see below), and the remaining sperm bundles were collected with a micropipette and diluted in a known volume of physiological solution to estimate the total amount of sperm produced by males at rest (hereafter ‘sperm number’). The sperm used for the velocity assay represented <3% of the total ejaculate, as the mean number of bundles per stripped male is >200 (C. Gasparini & A. Pilastro, unpubl. obs.). Sperm bundles were then broken by vortexing samples for about 1 min, and free sperm were counted using an ‘improved Neubauer’ haematocytometer (see Gasparini et al. 2010a). For the condition dependence experiment, we estimated the number of sperm by multiplying the number of sperm bundles stripped for the mean number of sperm per bundle estimated for this population (21,947 ± 1229 SE, *n* = 17), as sperm number and number of sperm bundles are highly correlated (*R*^2^ = 0.96, *P* < 0.0001, *n* = 17).

Sperm velocity was analyzed immediately after sperm collection, by adding an activating solution of 150 mmol/L KCl and 4 mg/mL bovine serum albumin to the sperm bundles and measuring the swimming velocity of the sperm moving away from the opening bundles (for details see Gasparini et al. 2009). We used a Hamilton-Thorne CEROS sperm tracker with the following parameters: frame rate 60 Hz; no. of frames 30; the threshold values defining static cells were predetermined at 20 *μ*m/s for VAP (see Gasparini et al. 2010a). Sperm velocity was estimated from a minimum of 100 sperm cell tracks. Two sperm velocity estimates were used: sperm path velocity (VAP, *μ*m/s), which is the average velocity of sperm cells over a smoothed cell path, and curvilinear velocity (VCL, *μ*m/s), which is the actual velocity along the trajectory. These measures provide an estimate of progressive velocity and have been shown to positively correlate with fertilization success in this guppy population (Boschetto et al. 2011) as well as in other poeciliids (Gasparini et al. 2010b). For each male, the motility analyses were performed on two subsamples of the same ejaculate and the mean was used in final analysis. In vitro measure of sperm velocity is repeatable in this population (Gasparini et al. 2009).

To measure sperm size, 50 *μ*L aliquots of each male's sperm sample used for estimating sperm number was incubated with 10 *μ*L of 1% Rose Bengal for 20 min. Dyed samples were then viewed under ×1000 magnification and photographed with a digital camera. Mean head length, midpiece length, and total sperm length (all measures in *μ*m) were obtained from >20 sperm per male using image analysis software (ImageJ).

A capture test, adapted from an established protocol used for newborn guppies (Evans and Magurran 2000; Evans et al. 2004), was used to estimate predator evasion capability of males, a condition-dependent trait (e.g., Grorud-Colvert and Sponaugle 2006) in the inbreeding (Exp. I) and the condition-dependent (Exp. II) experiments. Before sperm collection, each male was put into a tank (40 × 29 × 31 cm) with multicolor gravel on the bottom, which was similar to the tanks in which fish were housed. After 10-min acclimatization, one of us, blind to treatment, captured the fish using a small hand net (7 × 10 cm). Capture procedure consisted of chasing the male with the net at a speed, which was kept as constant as possible. The test started when the fish was in a central position in the tank, and the time until the fish was captured was recorded by another observer using a chronometer. If the male positioned himself against one of the tank glass walls, the net was held perpendicular to the glass and moved toward the fish (i.e., we avoided to capture the fish against the tank wall).

Repeatability in capture time was estimated from two capture sessions held, 1 day apart, on 19 individuals from Exp. I (9 inbred and 10 outbred, see below). Testing order within day was randomized, and capture tests were performed blind of inbreeding treatment and individual identity. Repeatability (intraclass correlation coefficient) was estimated following Lessells and Boag (1987), and SE of the estimate was calculated according to Becker (1984). Capture test showed a significant repeatability within individual (*R* = 0.68 ± 0.13 SE, *F*_18,37_ = 5.30, *P* < 0.001). This effect was not due to the difference in the mean capture time between groups, as repeatability did not change substantially when outbred and inbred males were analyzed separately (outbred: *R* = 0.63 ± 0.20 SE, *F*_9,19_ = 4.40, *P* = 0.015; inbred: *R* = 0.66 ± 0.20 SE, *F*_8,17_ = 5.00, *P* = 0.014).

#### Statistical methods

The data from experiments I and II were checked for normality of distribution and homogeneity of the variance. When these assumptions were not met, appropriate transformation was used. We used Student's *t-*test to compare means between groups. To control for body size-related effect, we used an ANCOVA in which the experimental group was the factor and the standard length was the covariate. The interaction between factor and covariate was used to test for the homogeneity of the slopes. Statistical tests were carried out using SPSS 18 (IBM Corporation, Software Group, Somers, NY) and GenStat 14 (VSN International Ltd, Hemel Hempstead, U.K.). If not otherwise specified, means ± SEs are given. Statistical methods for quantitative genetic analyses are given in detail below.

### Experimental design

#### Exp. I: Inbreeding depression

We estimated the inbreeding depression in second-generation males derived from full-inbred and outbred pairing. We artificially inseminated full-sib virgin females with sperm of a full-sib brothers or an unrelated male for two generations (inbreeding coefficient, *f* = 0.375) and obtained 33 families (17 inbred and 16 outbred). We randomly selected two 4-month-old sons from each family, even in a few instances we obtained only one son per family. The final number of males measured in the two groups was 30 (from 17 inbred families) and 29 (from 16 outbred families). Sperm number, sperm size, and sperm velocity were estimated as above. Inbreeding depression was calculated as the (*M*_O_−*M*_I_)/*M*_O_ × 100, where *M*_O_ and *M*_I_ were the mean phenotype of the outbred and inbred groups, respectively. We used a linear mixed model in which inbreeding was the fixed factor and family the random factor to account for nonindependence of males from the same family. Two weeks after sperm collection, males (one randomly chosen male per family) were tested in the simulated predator evasion test as described above.

#### Exp. II: Condition dependence

We used 84 6-month-old (range 5–7) male guppies that before the experiment were maintained in mixed-sex stock aquaria. Males, randomly assigned one of the two dietary treatments (ad libitum (nonrestricted), *n* = 42, and restricted, *n* = 42) were anaesthetized, weighted to the nearest 0.1 mg, digitally photographed and placed individually into 2-L tanks. Body mass and standard length did not differ between groups at the beginning of the experiment (SL: *t*_82_ = 0.022, *P* = 0.98; body mass: *t*_82_ = 0.516, *P* = 0.61). Ad libitum males received ca. 160 fresh *Artemia* nauplii twice a day, a number of nauplii exceeding those that are usually eaten by a male in 10 min, whereas males assigned to the restricted food group received one-quarter of this quantity. This diet restriction was slightly more severe than that adopted in a recent study which failed to show any effect on sperm number, velocity, and size (Devigili et al. 2013). Concentrated fresh *Artemia* nauplii were prepared each day. Their concentration was assessed using a haemocytometer-like slide, and an adjusted volume of the solution containing the nauplii was collected with a micropipette. The diet treatment lasted approximately 6 weeks (40.54 days ± 5.06 SD) and did not differ significantly in duration between groups (Student's *t*-test: *t*_72_ = 0.705, *P* = 0.482). At the end of the diet treatment, males were subjected to the capture test (see above), weighted to the nearest 0.1 mg, digitally photographed and stripped for ejaculate analyses (see above). Although we observed a higher mortality in the restricted group (8/42) as compared to the ad libitum group (2/42), the difference was not statistically significant (Fisher exact test, *P* = 0.09). Initial SL and body mass of males did not differ between the males that died during the treatment and those that survived (SL: *t*_72_ = 0.764, *P* = 0.45; body mass: *t*_72_ = 0.801, *P* = 0.43), and the experimental groups did not vary in their initial SL and body mass after excluding these males (SL: *t*_72_ = 0.516, *P* = 0.54; body mass: *t*_72_ = 0.022, *P* = 0.98).

Two males did not respond to the predator test and other two did not produce sperm (all from the restricted diet group). The bundles of five males (restricted group) did not open, and sperm velocity was thus not measured. We were unable to evaluate sperm morphology in 8 males (2 from ad libitum and 6 from restricted diet group) because of the insufficient number of photographed sperm (see Results for group sample sizes). Condition dependence was calculated as the (*M*_AL_−*M*_R_)/*M*_AL_ × 100, where *M*_AL_ and *M*_R_ were the mean trait value of ad libitum and restricted diet groups, respectively.

#### Exp. III: Quantitative genetic analysis

We used artificial insemination (Clark 1950; Evans et al. 2003) to inseminate virgin females to control for possible differential maternal allocation in relation to male phenotype, which may inflate sire heritability estimates (Kotiaho et al. 2003). Artificial insemination effectively excludes such effects as females were unaware of the identity of the males used as sperm donors. We randomly selected 66 mature males (sires) from our stock populations to inseminate 4–6 randomly assigned virgin females (dams) each. Sires were aged between 4 and 6 months and exhibited a range of color phenotypes. Following the same methods described above, sperm were manually stripped from males that were previously anesthetized. Once sperm bundles were obtained, females were anesthetized and placed in a polystyrene cradle with their genital pores exposed. A Drummond 3 *μ*L micropipette was used to inseminate 20 sperm bundles, suspended in 3 *μ*L of 0.9% NaCl, into each female's gonoduct. After insemination, females were revived in a 5-L plastic container where they remained isolated until they produced their first broods. Immediately after birth, the offspring were isolated from the mother in 5 L containers until they could be sexed. Each container contained gravel, natural, and artificial weed and an airstone. After offspring were sexed, males from each brood were distributed among 1–3 containers (1–2 males per container, total no. of containers = 318; mean number of male offspring per container = 1.81 ± 0.72 SD) and reared until sexually mature. In total, we obtained 574 sexually mature male offspring from 66 sires (mean number of male offspring per male = 8.70 ± 5.35 SD) and 145 dams (mean number of dams per sire = 2.20 ± 0.88 SD, range = 1–5; mean number of male offspring per dam = 3.96 ± 2.38 SD). Due to logistic constraints, the artificial inseminations were carried out in three temporally separated groups. In the first block of full-sib/half-sib families, we measured male body size at maturity and sperm size (sires = 33, *n* = 273), whereas in the second block of full-sib/half-sib families, we measured male body size at maturity (sires = 33, *n* = 176), sperm velocity (sires = 33; *n* = 176), and ejaculate size (sires = 33; sperm number at rest, *n* = 170).

Our nested half-sib breeding design enabled us to estimate additive genetic variance components (*V*_A_) required for the calculation of heritabilities (*h*^2^) and standardized coefficients of genetic variation (CV_A_). As guppies are viviparous, and several offspring are produced in the same brood, estimates of dam variance components are likely to be inflated by maternal effects and by common gestational and early life environment. The dam variance component and its associated genetic parameters have therefore to be interpreted cautiously. By contrast, the lack of paternal contribution to offspring (other than sperm), coupled with the use of artificial insemination (which prevents differential maternal allocation – see above), provide unbiased estimates of additive genetic variance (apart from *Y*-linked traits, see below), which forms the focus of the current study. The comparison between sire and dam heritabilities is nevertheless useful, because if narrow-sense heritability estimates attributable to sires exceeds those attributable to dams, *Y*-inheritance may be involved (Falconer and Mackay 1996; Brooks and Endler 2001a).

Brood size varied across sires and dams, and the statistical design was therefore unbalanced. Consequently, analyses of genetic variance components were conducted using restricted maximum likelihood (REML, Lynch and Walsh 1998). Genetic parameters (heritability and genetic correlations) and their associated SE were estimated using ASReml 3.0 (Gilmour et al. 2009). We conducted univariate linear mixed-effects models (with sire and dam entered as random effects) for each trait considered. From the variance components, we derived the additive genetic variances (*V*_A_) required for the estimation of narrow-sense heritability, using standard formulae for half-sib designs (four times the variance component due to sire, Falconer and Mackay 1996). The significance of the variance components was estimated using a likelihood ratio test (Lynch and Walsh 1998). In particular, we calculated twice the difference in log-likelihoods between full model and the model from which the sire (or dam) term was removed. The resultant goodness-of-fit statistic (*G*) approximates a chi-squared distribution with df = 1, corresponding to the number of parameters removed from the model (Lynch and Walsh 1998). Coefficients of additive genetic variation (CV_A_) were calculated following Houle (1992) from the formula 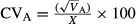, where *X* is the trait phenotypic mean.

We estimated the genetic correlation between sperm number and velocity (the two ejaculate traits more strongly associated with postcopulatory fitness), and among sperm size components, using the covariances owing to sires, as dam estimates are potentially inflated by common environmental and maternal effects. Genetic correlations and their standard errors were estimated in ASReml using a bivariate model with an unstructured variance matrix (Gilmour et al. 2009). To test the significance of each genetic correlation, we compared the full model with an unfixed covariance structure with that in which the covariance due to sires was fixed at zero. We then calculated twice the difference in log-likelihoods between fixed and unfixed models and tested this statistic against a chi-squared distribution with df = 1.

## Results

### Exp. I. Inbreeding depression

We found significant inbreeding depression in sperm number, whereas sperm velocity and size did not significantly differ between inbred males and their outbred counterparts (Table 1). Inbreeding depression in sperm number was not due to a smaller body size of inbred males, as body size did not show inbreeding depression and ID in sperm number remained significant after entering body size into the model as a covariate (*F*_1,25.6_ = 4.56, *P* = 0.043). Capture test revealed a large inbreeding depression in males' capability to evade a simulated predator (Table 1).

**Table 1:** Inbreeding depression (*δ*) in body size, ejaculate traits and condition (capture time). Traits that showed significant inbreeding depression are in bold. Means ± SE (*n*) are given. Differences between means were tested using a LMM in which female identity was entered as random factor, and inbred/outbred as fixed factor (significant differences in bold), with the exception of capture test, for which one randomly selected male per family was used.

Trait	Inbred	Outbred	Statistic	D df	*P*	*δ* (%)
Body size (SL, mm)	15.88 ± 0.19 (30)	16.05 ± 0.20 (29)	*F* = 0.15	30.8	0.70	1.1
Sperm number (×10^6^)	**4.24 ± 0.63 (30)**	**6.59 ± 0.73 (29)**	***F*** **=** **5.31**^1^	**23.6**	**0.03**	35.7
Sperm velocity (VAP, *μ*m/s)	103.63 ± 2.53 (25)	102.13 ± 1.90 (28)	*F* = 0.24	19.8	0.63	−1.5
Sperm velocity (VCL, *μ*m/s)	128.25 ± 1.96 (25)	128.29 ± 1.48 (28)	*F* = 0.01	23.7	0.93	0.0
Sperm head length (*μ*m)	4.16 ± 0.03 (25)	4.21 ± 0.02 (28)	*F* = 2.06	25.3	0.16	1.2
Sperm midpiece length (*μ*m)	4.64 ± 0.07 (25)	4.58 ± 0.08 (28)	*F* = 0.14	27.8	0.71	−1.3
Total sperm length (*μ*m)	55.24 ± 0.23 (25)	54.65 ± 0.28 (28)	*F* = 1.83	28.2	0.19	−1.1
Capture time (*s*)	**6.92 ± 1.92 (17)**	**14.4 ± 3.18 (16)**	***t*** **=** **2.47**^2^	**31**	**0.02**	**51.9**

1 Male body size (SL) entered as covariate.

2 Log-transformed before analysis; one male per family was used (outbred, *n* = 17; inbred, *n* = 16).

### Exp. II. Condition dependence

Our diet treatment significantly affected condition of male guppies with limited access to food, as indicated by a significant reduction in body mass (mean body mass: 62.84 mg ± 2.63 SE, *n* = 34) as compared to their ad libitum counterparts (75.84 mg ± 1.88 SE, *n* = 40; *t*_72_ = 4.103, *P* < 0.001). Diet-restricted males showed a significant reduction in sperm number, whereas sperm velocity and size were not affected by diet treatment, suggesting higher condition dependence in sperm production than in sperm quality (Table 2). Controlling for body size (SL) did not change this conclusion (data not shown).

**Table 2:** Effect of diet restriction on body size, ejaculate traits, and predator evasion capability (capture time). Mean ± SE (*n*) are given (significant differences in bold, Student's *t*-test). Condition dependence (CD, last column) was calculated as (*M*_AL_−*M*_R_)/*M*_AL_ × 100, where *M*_AL_ and *M*_R_ were the mean trait value of the ad libitum and restricted diet groups, respectively.

Trait	Ad libitum	Restricted	*t*	*P*	CD
Body size (SL, mm)	16.33 ± 0.13 (40)	15.97 ± 0.15 (34)	1.83	0.072	2.2
Sperm number (×10^6^)	**10.9 ± 0.69 (40)**	**7.82 ± 0.95 (34)**	**2.684**	**0.009**	28.3
Sperm velocity (VAP, *μ*m/s)	101.94 ± 1.38 (40)	100.37 ± 2.65 (27)	0.57	0.571	1.5
Sperm velocity (VCL, *μ*m/s)	126.31 ± 1.31 (40)	124.92 ± 2.3 (27)	0.566	0.573	1.1
Sperm head length (*μ*m)	3.94 ± 0.02 (38)	3.93 ± 0.04 (26)	0.349	0.728	0.3
Sperm midpiece length (*μ*m)	2.98 ± 0.04 (38)	2.99 ± 0.06 (26)	0.083	0.934	−0.3
Total sperm length (*μ*m)	55.91 ± 0.19 (38)	56.5 ± 0.29 (26)	1.779	0.080	−1.1
Capture Time (*s*)	**21.18 ± 2.51 (40)**	**14.2 ± 2.54 (32)**	**2.416**^1^	**0.018**	33.0

1 After log-transformation.

### Experiment III. Quantitative genetic analysis

Results of the quantitative genetic analysis are presented on Table 3. Sperm head and midpiece length, but not total sperm length, showed significant sire heritability. However, the observed coefficients of additive genetic variation were relatively low. Sperm velocity traits (VAP and VCL) did not show significant sire heritability and moderate to low coefficients of additive genetic variance. In contrast, the number of sperm stripped at rest exhibited a sire heritability estimate above 1 (suggesting *Y*-linkage) and a very large coefficient of additive genetic variation. This variation in sperm number was independent of variation in body size, as we did not find significant sire additive genetic variance for body size and estimates remained high after statistically controlling for differences in body size. We found significant dam effects for midpiece and total sperm length, while no significant dam effect on sperm number was detected.

**Table 3:** Observed coefficients of additive genetic and phenotypic variance and corresponding heritability estimates for body size and sperm traits. The variance components of pre- and postcopulatory traits were estimated from models with unconstrained variance structures. Heritabilities (with standard errors) due to sires (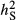) and dams (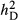) along with their associated *P*-values are indicated for each trait (NE = not estimable) (significant sire effects in bold). Estimates of coefficients (%) of phenotypic (CV_P_) and additive genetic variance for sire (CV_S_) are also reported.

Trait	Mean	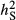 (SE)	*P* ( 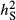 )	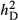 (SE)	*P* ( 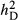 )	*N*_sire_	*N*_dam_	*N*_sons_	CV_P_	CV_S_
Body size (SL, mm)	17.85	0.41 (0.26)	0.08	1.07 (0.28)	<0.001	66	141	503	5.7	3.6
Sperm number (×10^6^)	**13.6**	**1.03 (0.34)**^2^	**0.005**	**0**^1^	**–**	**33**	**62**	**176**	**59.4**	**60.4**
Sperm velocity (VAP, *μ*m/s)	104.2	0.28 (0.33)	0.36	0.42 (0.42)	0.26	33	63	176	17.3	9.2
Sperm velocity (VCL, *μ*m/s)	128.4	0.44 (0.36)	0.19	0.49 (0.42)	0.17	33	63	176	13.0	8.7
Sperm head length (*μ*m)	**4.14**	**0.80 (0.34)**	**0.007**	**0.42 (0.28)**	**0.07**	**33**	**73**	**272**	**2.0**	**1.8**
Sperm midpiece length (*μ*m)	**5.39**	**0.95 (0.34)**	**<0.001**	**0.46 (0.34)**	**0.03**	**33**	**73**	**272**	**6.3**	**6.1**
Total sperm length (*μ*m)	53.64	0^1^	–	1.15 (0.29)	<0.001	33	73	272	1.1	0

1 As bounded REML method was used, negative variance components were forced to be 0 and SE cannot be calculated.

2 Male body size (SL) entered as covariate.

**Figure 2:**
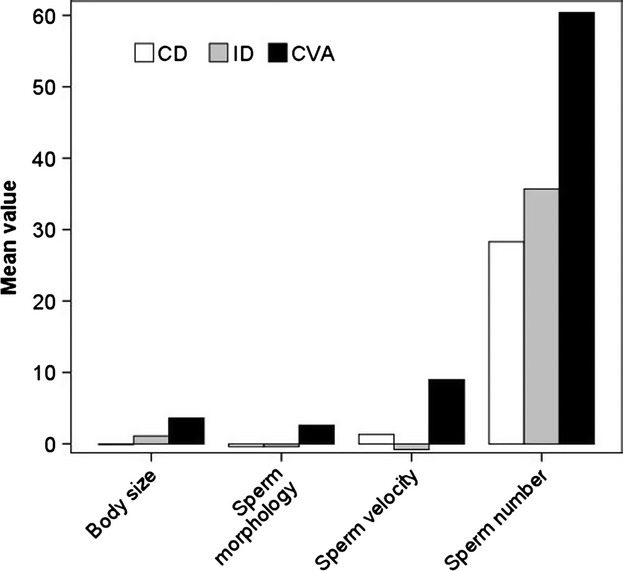
Inbreeding depression (ID), condition dependence (CD, see Table 2) and coefficient of additive genetic variation due to sire (CVA) in body size (standard length), sperm morphology (mean value of sperm head, sperm midpiece and total sperm length), sperm velocity (mean of VAP and VCL), and sperm number.

In contrast, dam heritability estimate for body length was larger than sire heritability and exceeded 0.5, suggesting maternal and/or early common environmental effects on male size at maturity. Overall, the pattern of variation in the coefficients of additive genetic variance across different ejaculate traits was remarkably congruent with the pattern of variation in inbreeding depression and condition dependence (Fig. 2).

We did not find significant genetic correlation between sperm number and velocity, nor between sperm velocity and different components of sperm size. In contrast, we found a significant negative genetic correlation between sperm head and midpiece length (Table 4).

**Table 4:** Phenotypic and genetic correlation among ejaculate traits. Observed phenotypic (above diagonal) and genetic (below diagonal) correlation coefficients with their standard error (within parentheses) among the sperm traits considered in this study. Significant genetic correlations (likelihood ratio test) and phenotypic correlations more than 1 SE away from zero are in bold. Genetic correlations between total sperm length and other traits were not estimable (the models did not converge).

	VAP	VCL	Head length	Midpiece length	Total sperm length	Sperm number
VAP	–	**0.89 (0.01)**	0.00 (0.09)	0.13 (0.09)	**0.17 (0.08)**	**0.19 (0.08)**
VCL	**0.97 (0.07)**	–	0.10 (0.08)	−0.09 (0.09)	0.01 (0.08)	**0.18 (0.08)**
Head length	−0.94 (0.62)	−0.49 (0.71)	–	−**0.27 (0.07)**	0.09 (0.07)	–
Midpiece length	2.50 (6.05)	−0.09 (0.63)	−**0.61 (0.25)**	–	**0.26 (0.07)**	–
Sperm number	0.77 (0.62)	0.53 (0.40)	–	–	–	–

## Discussion

The results of our inbreeding depression experiment confirmed previous evidence from an artificial insemination study that, in this guppy population, sperm number has been historically exposed to strong, directional selection, whereas sperm velocity and size were subject to weaker (sperm velocity) and probably stabilizing selection (sperm size components) (Boschetto et al. 2011). Inbreeding depression in sperm number was paralleled by a comparable reduction in the predator evasion test, a trait that is likely to be subject to directional selection in our population, which is collected in a high-predation site in Trinidad. Traits subject to strong, directional selection should show stronger condition dependence as compared to other traits that are less tightly associated with fitness (Pomiankowski 1987; Grafen 1990; Iwasa et al. 1991). Our diet experiment confirmed that unfavorable nutritional conditions have a larger effect on sperm number as compared to the other ejaculate traits and comparable with the reduced performance in predator escaping capability. Finally, stronger condition dependence should result into larger additive genetic variance (Rowe and Houle 1996; Houle 1998; Tomkins et al. 2004), although other mechanisms (see also below) can contribute maintaining large genetic variance in sexually selected traits (Walsh and Blows 2009). Again, our quantitative genetic estimates conform to this prediction (Fig. 2). We will discuss in more detail the results of our three experiments in succession.

The stronger inbreeding depression in sperm number that we observed was not a side effect of variation in body size, which was not affected by two generations of full-sib matings. Our result is in agreement with the previous work on a feral (Australian) guppy population, in which significant inbreeding depression in sperm production was found after two generations of full-sib matings (Zajitschek and Brooks 2010). Another study on the same guppy population revealed that sperm competitiveness is reduced only after four generations of full-sib mating when the number of sperm inseminated is experimentally controlled for using artificial insemination (Zajitschek et al. 2009). These two results indirectly suggest that the pattern of directional selection for sperm number and velocity may be similar in our and in the Australian population studied by Zajitschek and colleagues (although sperm quality has not been directly measured in the latter). In other species, inbreeding and homozygosity have been found to depress sperm quality (Fitzpatrick and Evans 2009) and sperm competition success (Konior et al. 2005; Michalczyk et al. 2010; Simmons 2011; Ala-Honkola et al. 2013). Although these latter studies did not identify the specific ejaculate trait showing inbreeding depression, they concur suggesting that postcopulatory performance is subject to directional selection.

When a sexual trait is subject to directional selection and it becomes exaggerated, it is expected to become costly (e.g., Garcia and Ramirez 2005). Indeed, theoretical models of sperm competition imply that sperm do have a cost and that resources that males use for spermatogenesis are limited (Parker 1970), an assumption for which there is also empirical evidence (e.g., Pitnick et al. 1995; Olsson et al. 1997; Simmons and Emlen 2006; Hayward and Gillooly 2011). For example, Japanese macaques (*Macaca fuscata*) use between 0.8% and 6.0% of their basal metabolic rate for ejaculate production per day during the breeding season, an estimate based on the energy content of ejaculates (Thomsen et al. 2006). Male guppies transfer a substantial part of their sperm reserves during a single mating (Pilastro and Bisazza 1999) and sperm reserves must therefore be replenished continuously, as male sexual activity is very intense (Magurran and Seghers 1994). Although the cost of sperm production has never been directly quantified in guppies, males adaptively tune their sperm investment according to mating opportunities (Bozynski and Liley 2003; Gasparini et al. 2009). Furthermore, here is experimental evidence that mating is associated with increased mortality in guppies (Miller and Brooks 2005).

If producing large numbers of sperm is costly and sperm number is under directional postcopulatory selection (Boschetto et al. 2011), sperm number is expected to evolve stronger condition dependence than sperm size and velocity (Pomiankowski 1987; Grafen 1990; Iwasa et al. 1991). Our diet restriction experiment had a significant effect on male guppy condition, as evidenced by the decrease in body mass and the significantly poorer performance in the evasion from a simulated predator (capture test). The reduction in condition associated with diet restriction was accompanied by a significant reduction in sperm number, whereas sperm velocity and size were not significantly affected. Collectively, these results clearly indicate a stronger linkage between condition and sperm production as compared to sperm quality traits, at least in this guppy population.

The results of our diet experiment are different from those obtained in two similar experiments conducted on a feral Australian guppy population. The first study did not evidence an effect of diet on sperm number, velocity, and size, although a reduction in sperm viability was observed (Devigili et al. 2013). In contrast, a more recent experiment on the same population (Rahman et al. 2013) evidenced a significant, negative effect of diet restriction on the all three ejaculate traits (sperm number, velocity, and size). Considering that different experimental manipulations are expected to have different effects on the same trait (Cotton et al. 2004), one most likely explanation for the different condition dependence pattern in these three studies is that the severity of our diet manipulation was intermediate between those used by Devigili et al. (2013) and Rahman et al. (2013). Indeed in Devigili et al. (2013), diet restriction lasted 1 month and restricted group received one-third of ad libitum's diet, whereas in Rahman et al. (2013), diet restriction lasted for 4 months. The interpretation of these results is further complicated because the Australian guppy populations have undergone recent bottlenecks following reintroduction, with significant loss of neutral genetic variability (Lindholm et al. 2005) and may not yet have reached equilibrium. Finally, it has to be noted that our diet restriction regarded only the total caloric content, whereas diet composition did not differ between treatments. Macronutrients deficits or diet composition, rather than caloric content, may affect sperm competitiveness, as it has reported in other organisms (e.g., Almbro et al. 2011). While this possibility cannot be ruled out, an experiment in which both diet caloric content and food composition were simultaneously manipulated revealed a very minor effect of the latter on ejaculate quality traits in guppies (Rahman et al. 2013).

Evidence of condition dependence of postcopulatory traits has also been found in other species. For example, in dung beetles *Onthophagus taurus*, testes size shows high coefficients of additive genetic variance and a positive genetic correlation with condition (Simmons and Kotiaho 2002). Interestingly, the effect of diet restriction on testes size is more evident in small, sneaker males, which face strong sperm competition and have large testes, than in large, fighting *O. taurus* males, which show, in contrast, a significant reduction in physical strength but not in testes size (Knell and Simmons 2010), suggesting that the pattern of condition dependence reflects the strength of sexual selection acting on these traits. Although we did not have direct evidence that sperm number is genetically correlated with condition, we found that both inbreeding and diet restriction negatively affected male performance (capture test) in a predator evasion test, a measure that has been related to genetic quality in guppies (Evans and Magurran 2000; Evans et al. 2004) and to condition in other fish species (Grorud-Colvert and Sponaugle 2006).

The pattern of condition dependence that we found in sperm number, velocity, and size was mirrored by the results of our quantitative genetic analysis, which revealed that sperm number had the largest sire CV_A_, while lower coefficients were associated with sperm velocity and size. Sperm number exhibited the highest value of sire heritability among the ejaculate traits we measured, in line with heritability patterns reported in other taxa for sperm production traits (Simmons and Moore 2009). In contrast, sperm head and midpiece, which also showed significant sire heritability, were characterized by lower coefficients of additive genetic variation, similarly to the results of previous studies in the Australian guppy population (Evans 2010). Although *Y*-linkage in sperm number may have inflated our estimate of the coefficient of additive genetic variation, it has to be noted that sperm head and midpiece also showed heritabilities close to one (i.e., suggesting a similar degree of *Y*-linkage), yet their CV_A_ was one order of magnitude smaller than that in sperm number. Furthermore, after correcting the estimate of additive genetic variation to account for *Y*-linkage (twice the variance component due to sire, Falconer and Mackay 1996), CV_A_ in sperm number would be 42.7%, still considerably larger than that (uncorrected) in sperm velocity. We can also exclude that the observed differences in CV_A_ between sperm traits were due to different scaling among traits (Garcia-Gonzalez et al. 2012), as sperm number, the trait with the highest CV_A_, was expressed at a smaller scale than both sperm total length and sperm velocity (Table 1). In agreement with empirical data, our results therefore confirm that the closer a trait is to fitness, the larger is its underlying genetic variance (Houle 1992, 1998).

Although our results collectively suggest that condition dependence, possibly through mutation-selection balance (Rowe and Houle 1996), may be responsible for the maintenance of genetic variability in sperm number, other mechanisms that might account for the observed pattern of genetic variance have been proposed (Johnson and Barton 2005; Radwan 2008). For example, it has been suggested that a large genetic basis for a trait inevitably leads to genetic correlation with other traits and pleiotropy, which will slow down the efficiency of selection in reducing the genetic variance in a single trait. Adaptation is an inherently multivariate process and the large genetic variance in a single fitness-related trait may be the signature of strong multivariate genetic constraints (Walsh and Blows 2009). The ejaculate is a multitrait, functionally and genetically integrated unit to ensure fertilization and increase male sperm competition success. Indeed, data from other quantitative genetic studies suggest that the evolution of an ejaculate's single trait can be constrained by negative genetic correlations with other sperm traits (Moore et al. 2004; Birkhead et al. 2005; Evans 2011; but see Thuler et al. 2011), and previous quantitative genetic studies on guppy revealed the existence of genetic constraints among sperm velocity and sperm size traits (Evans 2011) and between these sperm traits and precopulatory traits (Evans 2010), although sperm number was not included in these studies. We also evidenced a genetic integration between sperm head and midpiece length, which may be the result of stabilizing selection on overall sperm size. In agreement with this conclusion, phenotypic variation in sperm size has no effect on sperm velocity (Simpson et al. 2013) and fertilization success (Boschetto et al. 2011), In contrast, the genetic correlation between sperm number and sperm velocity was positive, although not statistically significant (Table 4). Furthermore, there is no evidence that sperm number is genetically, negatively correlated with traits associated with mate acquisition (relative area of orange spots, *r* = −0.26 ± 0.31 SE, iridescent spots, *r* = 0.27 ± 0.26 SE; body length, *r* = 0.77 ± 0.89 SE; C. Gasparini and A. Pilastro, unpubl. results). Indeed, an ongoing experiment of artificial selection for sperm number revealed that this trait responds readily to directional selection and that males producing large sperm number show simultaneous null or positive correlated responses by other sexually selected traits (A. Di Nisio and A. Pilastro, unpubl. obs.). Genetic constraints due to correlations with other genes and gene networks are expected to be attenuated, if a trait, such as sperm number, is subject to continuous strong selection for a sufficient number of generations. Indeed, Trinidadian guppies have likely a long evolutionary history of sperm competition, as very high levels of multiple paternity are found in all Trinidadian guppy populations, and are typical of this fish family (Evans and Pilastro 2011). Our knowledge of the competitive fertilization process is not complete, though. For example, postcopulatory fitness surfaces may be more complex when long-term sperm storage is taken into account (Orr and Zuk 2012). Indeed, there is evidence from another poeciliid species that sperm velocity is negatively correlated with sperm competition success when sperm are stored in the female for long times (Smith 2012). Guppies store sperm for months, but the association between ejaculate characteristics and fertilization success after long-term storage has not been investigated so far. Clearly, more work is necessary to clarify these aspects.

Considering that increased sperm production is the typical evolutionary response to increased levels of sperm competition, at least in vertebrates (Immler et al. 2011), it may be predicted that the corresponding pattern of condition dependence and genetic variance should be larger for sperm production than for other sperm traits also in other species with high levels of sperm competition. Interspecific comparison suggests that additive genetic variation may be relatively larger for sperm production traits (see Fig. 1 in Simmons and Moore 2009). It seems interesting, however, that this pattern is reversed in *Drosophila pseudoobscura*, in which sperm size shows significant, although moderate, CV_A_ (3–7%), whereas CV_A_ in sperm production is negligible (<0.2%, Snook et al. 2010), if one considers that sperm size positively affects sperm competition success in *Drosophila* (Lüpold et al. 2012). Comparative analysis may help drawing general conclusions about the pattern of genetic variation underlying sperm production versus sperm quality traits, once data for a larger number of taxa will become available.

In conclusion, our results provide explicit evidence that differential selection pressures among sperm traits predicts their pattern of additive genetic variation and condition dependence, as expected under a ‘“good genes” process at the postcopulatory level (Yasui 1997). When sire condition is correlated with his fertilization success (mediated by sperm number), females mating promiscuously will benefit of an increased offspring survival and an enhanced reproductive success of their sons. Our results indicate that sperm number, through its association with overall male condition and its large additive genetic variance, can fuel polyandry in guppies and provide a mechanism explaining the genetic benefits associated with multiple matings in this species (Evans and Magurran 2000; Ojanguren et al. 2005; Barbosa et al. 2012). Further work will be necessary to clarify the contribution of other mechanisms to the maintenance of genetic variation in sperm number and hence the nature of the genetic benefits associated with female multiple mating. For example, the relative contribution of loci with intermediated allele frequency versus partially recessive deleterious mutations at low frequency to the observed genetic variance in sperm number is still unknown. The comparison of the response to artificial selection for high and low sperm number with the inbreeding depression in the selected lines (Kelly 1999) will allow to directly test the assumption that the expression of condition-dependent, sexually selected, traits reflects the mutational load of males (Rowe and Houle 1996).
